# Alterations of the nigrostriatal pathway in a 6-OHDA rat model of Parkinson’s disease evaluated with multimodal MRI

**DOI:** 10.1371/journal.pone.0202597

**Published:** 2018-09-06

**Authors:** Vincent Perlbarg, Justine Lambert, Benjamin Butler, Mehdi Felfli, Romain Valabrègue, Anne-Laure Privat, Stéphane Lehéricy, Alexandra Petiet

**Affiliations:** 1 UPMC / INSERM UMR975, Brain and Spine Institute, Paris, France; 2 Bioinformatics and Biostatistics Core Facility, Brain and Spine Institute, Paris, France; 3 Center for Neuroimaging Research, Brain and Spine Institute, Paris, France; Hokkaido Daigaku, JAPAN

## Abstract

Parkinson’s disease is characterized by neurodegeneration of the dopaminergic neurons in the substantia nigra pars compacta. The 6-hydroxydopamine (6-OHDA) rat model has been used to study neurodegeneration in the nigro-striatal dopaminergic system. The goal of this study was to evaluate the reliability of diffusion MRI and resting-state functional MRI biomarkers in monitoring neurodegeneration in the 6-OHDA rat model assessed by quantitative histology. We performed a unilateral injection of 6-OHDA in the striatum of Sprague Dawley rats to produce retrograde degeneration of the dopamine neurons in the substantia nigra pars compacta. We carried out a longitudinal study with a multi-modal approach combining structural and functional MRI together with quantitative histological validation to follow the effects of the lesion. Functional and structural connectivity were assessed in the brain of 6-OHDA rats and sham rats (NaCl injection) at 3 and 6 weeks post-lesioning using resting-state functional MRI and diffusion-weighted. Our results showed (i) increased functional connectivity in ipsi- and contra-lesioned regions of the cortico-basal ganglia network pathway including the motor cortex, the globus pallidus, and the striatum regions at 3 weeks; (ii) increased fractional anisotropy (FA) in the ipsi- and contralateral striatum of the 6-OHDA group at 3 weeks, and increased axial diffusivity (AD) and mean diffusivity in the ipsilateral striatum at 6 weeks; (iii) a trend for increased FA in both substantia nigra of the 6-OHDA group at 3 weeks. Optical density measurements of tyrosine-hydroxylase (TH) staining of the striatum showed good correlations with the FA and AD measurements in the striatum. No correlations were found between the number of TH-stained dopaminergic neurons and MRI measurements in the substantia nigra. This study suggested that (i) FA and AD were reliable biomarkers to evaluate neurodegeneration in the cortico-basal ganglia network of the 6-OHDA model, (ii) diffusion MRI and resting-state functional MRI (rsfMRI) were not sensitive enough to detect changes in the substantia nigra in this model.

## Introduction

Parkinson’s disease (PD) is a major neurodegenerative disease in the elderly affecting 10 million patients worldwide (according to the Parkinson’s Disease Foundation). PD is characterized by massive degeneration of the dopaminergic neurons in the substantia nigra pars compacta (SNc), leading to clinical symptoms such as tremor, bradykinesia, and rigidity (according to the Parkinson’s Disease Foundation). It is generally accepted that PD occurs when already 50% of those neurons are destroyed [1]. Non-invasive imaging biomarkers may be used for early diagnosis and monitoring of drug effect and therefore for the development of more efficient treatments against the disease. Structural and functional MRI biomarkers have been developed in PD and offer the opportunity to explore brain functional and anatomical connectivity changes [2]. However, to date, histological validation of these markers is still lacking. In this regard, animal models play a critical role for the evaluation of biomarkers that can monitor disease progression and the effects of new treatments.

Indeed, several imaging approaches, including MRI, have been employed to investigate brain degenerative processes [3]. Among them, diffusion tensor imaging (DTI) has been used to assess changes in microstructure structures within brain tissues in both humans and animals. In particular, DTI has shown neurodegeneration in the SN and nigro-striatal fibers in PD patients with reduced fractional anisotropy in the SN [4–7] and reduced SN structural connectivity [8–10], confirming that efforts must be made in diffusion imaging markers evaluation and selection in combination with brain histology in animal models.

Resting-state functional MRI (rs-fMRI) is a promising MRI technique that has the potential to detect changes in the functional properties of these structures [11,12]. Reduced functional connectivity (FC) of the SN was reported in patients [9,13,14]. However, the histological correlates in animal models of these changes in connectivity are poorly understood. For the last decade, rs-fMRI has been extended from humans to animals to non-invasively study FC of the brain [15–17]. Studies in rats have shown consistent resting-state correlations maps with known functional pathways [16]. A recent study reported similar and robust resting-state networks in rat and human brains, inferring that this method can be used as a valuable tool for translational research in brain disorders [18].

Various 6-hydroxydopamine (6-OHDA) rat models have been developed in which this toxin was injected into different parts of the nigrostriatal pathway. The most common model involves a unilateral injection of 6-OHDA into the SN or into the medial forebrain bundle (MFB), leading to rapid cell death. Another model involves injection of 6-OHDA directly into the striatum (STR) [19,20], causing slower retrograde degeneration of up to 70% of the dopaminergic neurons in 4 weeks [21], thereby allowing longitudinal follow up of the lesion in a more progressive manner. This model is then well designed to study connectivity alterations.

The main goal of our study was to investigate the structural and functional connectivity alterations caused by 6-OHDA striatal lesions with a multi-modal approach, and to study the histological correlates of MRI changes.

## Material and methods

### Animals

All animal experiments were performed in accordance with the EU Directive 2010/63/EU for animal experiments and approved by the Charles Darwin's Ethical Committee for Animal Experimentation (Ce5/2011/050).

Fifteen Sprague Dawley male rats (300–450g) were used in this study. For stereotaxic surgery, the animals were first anesthetized with Isoflurane (3% then 0.75%, Abbott Animal Health) followed by an intra-muscular injection of a Ketamine/Xylazine mixture (Imalgene 500 at 50 mg/kg, Merial, and Rompun^**®**^ 2% at 10 mg/kg, Bayer Animal Health). Ten rats were injected with 2 μl per injection site of 6-OHDA (4.86 mg/ml of 6-OHDA in ascorbic acid at 0.01% and NaCl 0.9%) and 5 sham rats were injected with a physiological solution (ascorbic acid at 0.01% and NaCl 0.9%). The two unilateral injections were localized in the right dorsal striatum at coordinates (A+0.5; L+2.5; P-5) and (A-0.5; L+4; P-5).

All rats were MRI scanned 3 weeks then 6 weeks after injection. The animals were fed *ad libidum* until one week after surgery. They were then put on a restricted diet to prevent them from gaining excessive weight (5 pellets per day), such that their weight remained under 450g at 6 weeks post-injection. All animals were weighed on a daily basis. During the MRI acquisitions, the animals were lightly anesthetized with 1.5% Isoflurane (Abbott Animal Health) mixed with oxygen and air (1:5 O_2_:air) delivered through a nose cone at a flow rate of 1 L/min. Physiological parameters were recorded via a monitoring system (S.A. Instruments Inc.). The temperature of the animals was maintained at 37°C via a circulating heated water system, and monitored via a rectal probe. The respiration of the animals was monitored via a pressure pad positioned under their abdomen.

### Data acquisition

All images were acquired with a 11.7-T system (Bruker Biospec 117/16 USR horizontal bore, 750mT/m gradients, Paravision 5.1, Ettlingen, Germany) and a surface receive-only coil for rat head for signal reception and a 72-mm resonator for signal emission (Bruker, Ettlingen, Germany). Local first and second order shimming was performed using a Bruker MAPSHIM macro based on a fieldmap acquisition (500μm isotropic resolution; first-TE = 1.4ms; delta-TE = 3.4ms). Anatomical T_2_-weighted (T_2_w) images were acquired with a multi-slice multi-echo (MSME) sequence and the following parameters: TR = 8000ms; TE = 13.5ms; Matrix (Mtx) = 384x256; field-of-view (FOV) = 3.84x2.56cm^2^; voxel size = 100x100μm^2^; slice thickness = 200μm; 128 slices; number of averages = 1; acquisition time (Tacq) = 34min. For rs-fMRI, multi-slice echo-planar images (EPI) were acquired with TR = 3000ms; TE = 17.5ms; receiver bandwidth = 400kHz; Mtx = 96x96; FOV = 3.84x3.84cm^2^; voxel size = 400x400μm^2^; slice thickness = 400μm; 52 slices; 110 repetitions; 4 dummy scans to allow T_1_ steady-state; Tacq = 5min30s. For diffusion-weighted imaging, 3-dimensional EPI were acquired with the following parameters: TR = 300ms; TE = 24ms; 81 gradient directions; b-value = 1500s/mm^2^; ∂ = 4ms; Δ = 10ms; voxel size = 250μm^3^ isovoxel; Tacq = 42min.

### MRI data analysis and statistics

First of all, a T_2_w brain template was created from all individual volumes. To do so, each T_2_w image was co-registered to the Karolinska rat brain template [22] in the Paxinos standard space by using Ants software then averaged across the individuals. Three rats and an additional one at 6 weeks were excluded from the analyses because of data quality issues.

Functional time-series were motion-corrected by rigid-body realignment to the first volume, spatially smoothed (Gaussian kernel with FWHM = 500μm) using SPM8 software and corrected from physiological noise by using the compCorr algorithm.

Regions-of-interest (ROIs) of the motor network were defined from two complementary atlases: a stereotaxic rat brain atlas derived from Paxinos atlas [7] for motor cortex (M1/M2) and somatosensory cortex (S1/S2) regions and the Waxholm space atlas [23] for subcortical regions including the dorsal striatum (STR), the SN [24], the thalamus (THA) [25] and the globus pallidus (GP). Striatum regions were manually corrected to remove the injection lines. ROIs were placed in the hemispheres ipsilateral (ipsilateral ROI, right) and contralateral (contralateral ROI, left) to the 6-OHDA injections.

To study the whole brain FC changes from STR seeds between the 6-OHDA and the sham groups, FC maps were calculated for each individual from the averaged time-course within left and right STR independently. Group differences (sham versus 6-OHDA at 3 weeks and at 6 weeks) were tested by using a two-sample unpaired t-test with family-wise error (FWE) rate controlled for multiple comparisons (p<0.05) and including the threshold-free cluster enhancement (TFCE) method for clusters detection.

Region-to-region FC was derived from averaged time-courses within each ROI of the motor network. Correlation coefficients were Z transformed before group comparison at 3 and 6 weeks, tested with a Wilcoxon ranksum test using false discovery rate (FDR) correction for multiple comparisons (p<0.05). As FC values from SN were extremely weak (around 0), SN regions were removed from further correlation analysis.

DWI data were preprocessed with standard tools of FSL 5.07 (FMRIB, Oxford, UK): eddy_cor for eddy current correction and fugue for epi distortion correction with the fieldmap acquisition. We then used dtifit to compute the tensor metrics. ROIs were manually drawn in the dorsal STR and in the SN (N = 7 6-OHDA at 3 weeks, N = 6 6-OHDA at 6 weeks and N = 5 sham rats) from the FA maps of the native space using MRView (MRtrix3 viewing tool, http://www.brain.org.au/software/). Care was taken to avoid the injection lines and to include comparable numbers of voxels within the ROIs across animals. The fiber tracking analysis was performed with the MRtrix3 package using deterministic tractography (http://www.mrtrix.org). Weighted means were calculated along the reconstructed tracks from the FA, RD, AD, MD maps.

Statistics were performed with R software (http://www.R-project.org). The two experimental groups were compared by using a non-parametric Wilcoxon ranksum tests. Results were considered significant for p<0.05.

### Histology

Seven weeks after injections, the rats were deeply anesthetized with an i.p. injection of pentobarbital (150mg/kg, Euthasol Vet^**®**^ 400 mg/ml) and perfused with 100 ml of saline solution to washout the blood, followed by 300 ml of 4% ice-cold paraformaldehyde (PFA) solution. The brains were dissected out and post-fixed in 4% PFA overnight at 4°C, cryoprotected in 30% sucrose solution for 48h, frozen in isopentane at -30°C and stored at -80°C. Horizontal 40μm-thick sections were cut with a freezing microtome.

For each brain, four anatomically-matched sections evenly spaced by 400-μm intervals covering the dorso-ventral extent of the structure were selected for quantification.

TH-positive (TH+) fibers in the dorsal STR were quantified with optical density (OD) measurements. The sections were digitized with an Epson scanner and the measurements done with an image analysis software (MCID Image Analysis Software Solutions for Life Sciences, Interfocus Imaging Ltd., Linton, UK). The STR was manually delineated from four sections per animal and the ratios between the OD of a reference structure with no TH staining (reference background measurement) and the OD of STR were calculated. The data are represented as the ratio STR/background (mean ± standard deviation, SD).

Stereological assessment of the number of dopaminergic neurons in the SNc of TH+ cell bodies was performed using an unbiased stereology method with a computer-based image analyzer (Mercator, ExploraNova, La Rochelle, France) as previously described [25]. The SNc was manually delineated in regularly spaced sections every 400 μm covering the dorso-ventral extent of the structure. The quantification relies on the use of unbiased stereology probes, the optical dissector and the fractionator sampling scheme [26]. Optical dissectors were distributed using a systematic sampling scheme. The mean thickness was about 19 μm. We used 60-μm long and 60-μm wide dissectors, with 75 μm × 75 μm vertical and horizontal separations. Neurons were counted as they came into focus within the frame. Only the neurons situated entirely within the optical dissector, or crossing two preselected dissector borders (acceptance lines) were counted [27].

All values are expressed as mean ± SD. All statistical analyses were conducted using R Statistical Software (version 3.3.3; R Foundation for Statistical Computing, Vienna, Austria). The non-parametric Mann-Whitney-Wilcoxon Test was used for two-group comparisons. Correlations were performed by Spearman's correlation coefficient, calculated between the MR values and the relevant OD measures. P-value < 0.05 was set as significance level.

## Results

### Functional connectivity

**Fig 1:**
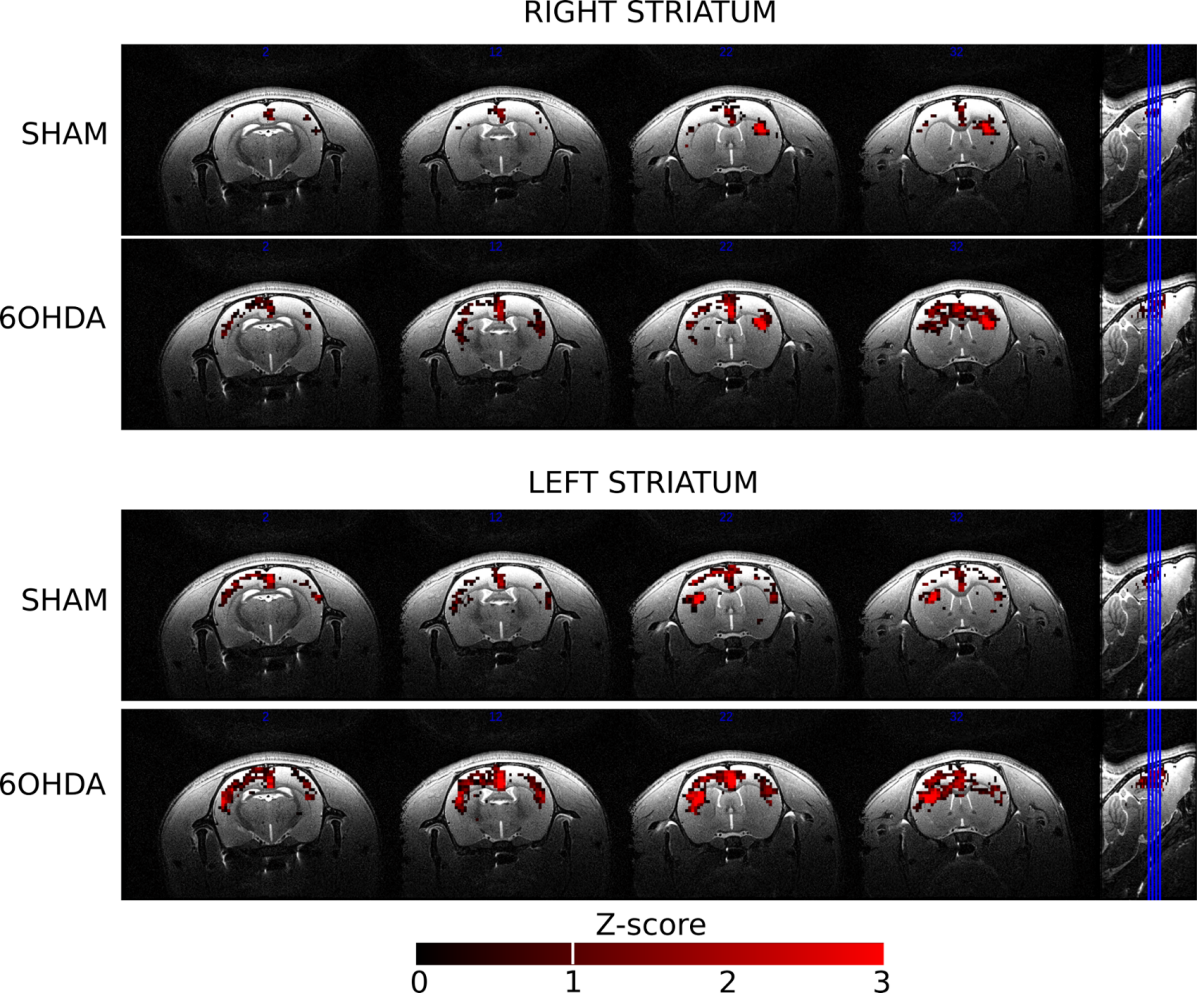
Group FC maps from left and right striatum for the sham and 6-OHDA groups. Maps are expressed as Z-scores. The 6-OHDA group showed larger bilateral patterns of FC than the sham group at 3 weeks but the difference was not significant.

Although FC maps suggested greater FC in 6-OHDA compared with sham animals (Fig 1), the difference did not reach significance and there were no significant voxel-wise differences in FC maps between sham and 6-OHDA groups at 3 weeks and 6 weeks.

**Fig 2:**
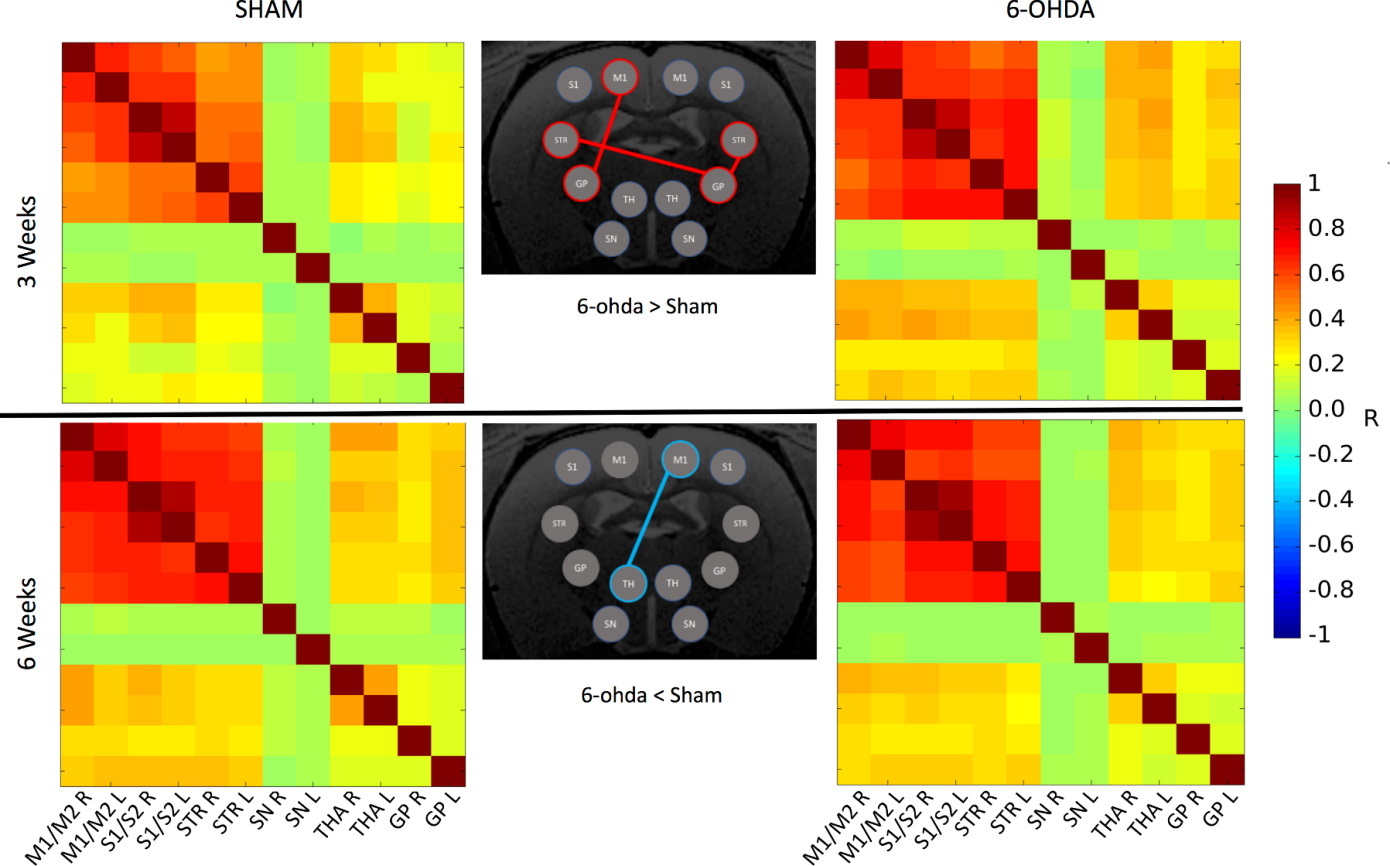
FC matrices within the motor network at 3 weeks and 6 weeks. Red (resp. Blue) links indicate significant positive (resp. negative) difference in FC between the 6-OHDA group and the sham group.

Connectivity matrices at 3 weeks showed increased FC in the 6-OHDA group compared to the sham group (Fig 2). At 3 weeks, significant increases of FC were found between ipsilateral GP and ipsilateral STR (p<0.05), between ipsilateral GP and contralateral STR (p<0.05) and between contralateral GP and contralateral M1/M2 (p<0.05). At 6 weeks, significant decrease in FC was found between contralateral THA and ipsilateral M1/M2 (p<0.05).

### Diffusion-weighted MRI

**Fig 3:**
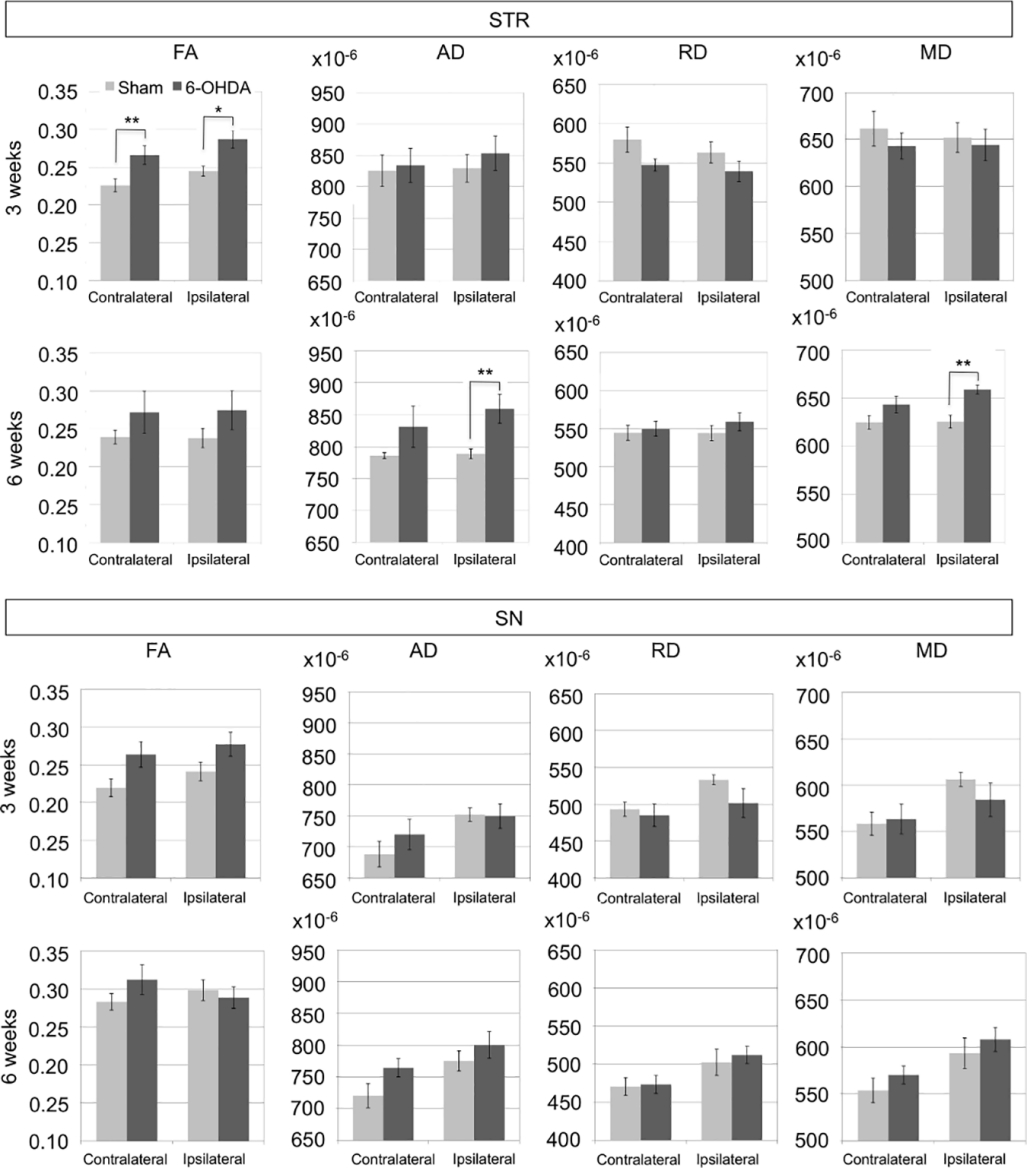
Diffusion metrics in the STR and the SN at 3 and 6 weeks. AD, RD, MD are in mm^2^/s. Significant results are indicated using *p<0.05 and **p<0.01.

At 3 weeks, there was increased FA in the ipsi- and contralateral STR (p<0.03 and p<0.005, respectively) in the 6-OHDA group compared to the sham group (Fig 3). At 6 weeks, AD and MD increased in the ipsilateral STR (p<0.01) of the 6-OHDA group. No significant differences in diffusion metrics between contralateral and ipsilateral sides were found. No significant differences were found in the SN (Fig 3).

### Histology

There was a significant decrease in OD measurements in the ipsilateral STR in the 6-OHDA group compared to the sham group (p<0.02), indicating striatal dopaminergic denervation. The number of neurons in the ipsilateral SNc significantly decreased by 60% in the 6-OHDA group compared to the sham group (mean ± SD: 2857 ± 980 vs. 6840 ± 1429 neurons in the 6-OHDA and sham groups respectively, p<0.05), confirming retrograde dopaminergic depletion after lesioning. Furthermore, we found a significant loss of 75% of TH-positive neurons in the ipsilateral compared to the contralateral SNc in the 6-OHDA group (mean ± SD: 2857 ± 980 vs. 11153 ± 1931 neurons in the ipsilateral and contralateral hemispheres respectively, p<0.0002).

There were significant differences in OD between the two hemispheres (p<0.003) and no significant differences between the contralateral hemispheres of the sham and 6-OHDA groups (p<0.9), which suggested no interhemispheric effects from the lesion. In addition, there were no significant differences between the ipsilateral and contralateral STR of the sham group (p = 1), which confirmed that saline injections did not induce any lesion in this region. Together those results indicated neuronal loss in the dopaminergic pathways exclusively in the ipsilesional hemisphere of the 6-OHDA rats.

### MRI-histology correlations

**Fig 4:**
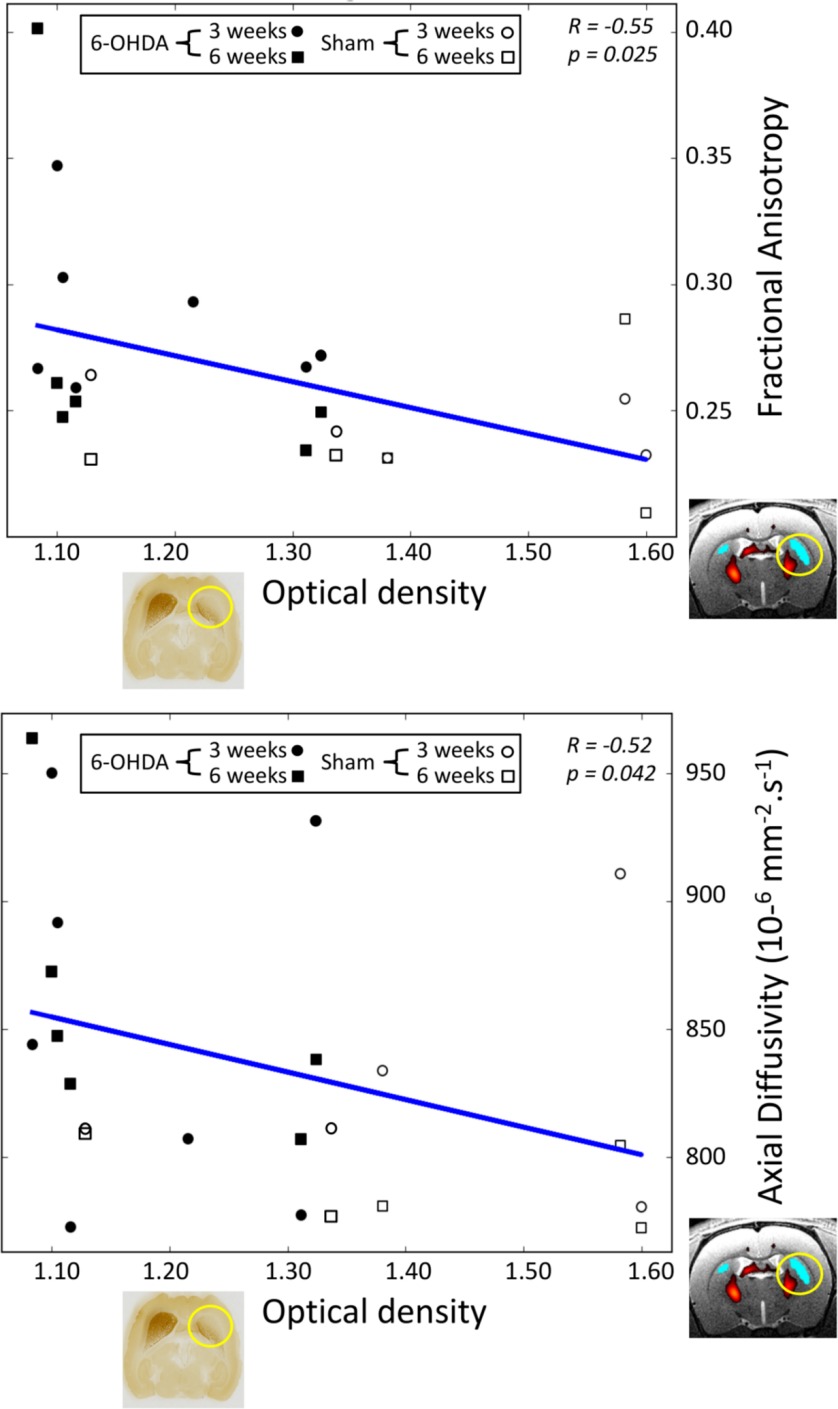
Scatter plot of fractional anisotropy and axial diffusivity versus optical density values. The diffusion values are measured within the right STR for all exams. Circles correspond to 3 weeks exams and squares to 6 weeks exams. Black markers stand for 6-OHDA group and white markers for sham group.

Correlation between MRI markers and OD measurements within STR were tested from all exams. There were significant correlations between dopaminergic degeneration (OD) in the ipsilesional STR in 6-OHDA rats and (i) the increase in FA (p< 0.03, R = -0.63) and (ii) the increase in AD (p<0.03, R = -0.62) in the STR (Fig 4). There were no other correlations for diffusion or FC changes.

## Discussion

Alterations in FC were found within (i) ipsilateral sub-cortical regions, which may reflect direct lesioning effects in those regions (STR and GP), (ii) contralateral cortical and sub-cortical regions (M1/M2 and GP) and (iii) interhemispheric subcortical regions (STR and GP), both of which may reflect compensatory effects. These results are in line with EEG studies in 6-OHDA rats, which have also shown increased functional coupling in basal ganglia including in STR-GP connections [28,29]. Westphal et al. [30] showed reduced FC within cortical regions, but bilaterally increased FC in subcortical regions such as the thalamus, although these results were found in a different rat model of PD (medial forebrain-lesioned 6-OHDA rats) [30]. In addition, Debeir et al. [31] used the same STR-lesioned rat model and showed loss of TH(+) fibers in the GP, which could explain MRI alterations in this structure [31]. No significant FC effects were found with the SN, presumably due to the small size of this structure, which led to lack of sensitivity in the image acquisition scheme and poor signal-to-noise ratio of the BOLD signal within SN.

Microstructural alterations were found in ipsilateral as well as contralateral STR. Increased FA and MD in the STR may be attributed to neurodegeneration processes in this region with crossing fibers [32,33]. Once again, results within SN did not reach significance. Alternative diffusion biomarkers could be considered to improve detections. Hence, free-water bi-tensor model showed increased effect size in diffusion changes detection within SN in PD patients [34].

Finally, the correlation between the dopaminergic depletion in the STR and increased FA and AD confirms that diffusion parameters are interesting biomarkers of neurodegeneration in this model. However we did not find any significant differences in the diffusion metrics between the ipsilateral and contralateral hemispheres in the 6-OHDA rats. This is presumably due to the lack of sensitivity of the diffusion method and the small sample size. At the opposite, no relationship was found between dopaminergic depletion in the STR and FC parameters. This observation as well as the relative discrepancy in rs-fMRI findings in the literature (including our study) suggests that rs-fMRI is not a good marker of neurodegeneration per se but only of the resulting functional changes. Several rs-fMRI studies have also been conducted in humans, showing decreased FC within the whole brain [35], in the motor system [14,36,37] including the striatum [13] and the SN [9] as well as increased FC between the striatum and the associative cortex [14], the subthalamic nucleus and midline motor areas [24] or both (increase and decrease FC) within nigro-striato-cortical networks [38]. Decreased and increased FC are often interpreted as pathological or compensatory processes respectively but clear evidences are needed. Even though recent progress has been made in functional connectivity in rodent [39], the variety of methodological approaches and the lack of reproducibility of results delay its larger implementation in basic and applied research.

Overall, animal models of PD recapitulate only partially the human pathology. The striatal 6-OHDA lesioning rat model, although the most « progressive » model compared to the more acute medial forebrain or SN injection models, is a poor representation of the human pathology as (i) the partial lesions occur within weeks only throughout the lifetime of the animal and (ii) the structural and functional MRI metrics are not modified the same way as in humans. Other studies have reported discrepancies between human pathology and animal models [40], so interpretation of the data should be done with caution and a better physio-pathological understanding of the model is required.

## Conclusions

This study showed that FA and AD changes in the striatum correlated with the histological reduction in dopaminergic terminals as assessed using TH optical density and hence may be reliable biomarkers to evaluate neurodegeneration in the nigrostriatal pathway of the 6-OHDA model. However, diffusion MRI and rsfMRI, with conventional acquisitions and analysis strategies, were not sensitive enough to detect any changes in the SN in this model. Advanced acquisition schemes and adapted data analysis are required to properly measure microstructural alterations and functional changes within SN in rodents. These advances should be accompanied by a better characterization of pathological processes in 6-OHDA (and other) PD models.
